# Correspondence: Osteonecrosis in childhood acute lymphoblastic leukemia: a retrospective cohort study of the Italian Association of Pediatric Haemato-Oncology (AIEOP)

**DOI:** 10.1038/s41408-018-0150-z

**Published:** 2018-11-15

**Authors:** Rosanna Parasole, Maria G. Valsecchi, Daniela Silvestri, Franco Locatelli, Elena Barisone, Fara Petruzziello, M. Caterina Putti, Concetta Micalizzi, Antonella Colombini, Rossella Mura, Tommaso Mina, Anna M. Testi, Lucia D. Notarangelo, Nicola Santoro, Tommaso Casini, Caterina Consarino, Luca Lo Nigro, Ottavio Ziino, Giovanna Giagnuolo, Carmelo Rizzari, Valentino Conter

**Affiliations:** 1 Department of Pediatric Hemato-Oncology, A.O.R.N. Santobono-Pausilipon, Naples, Italy; 2 Center of Biostatistics for Clinical Epidemiology, Department of Health Science, University of Milano-Bicocca, Milan, Italy; 3 Pediatric Hematology-Oncology Unit, Department of Pediatrics, University of Milano-Bicocca, MBBM Foundation/ASST Monza, Monza, Italy; 4 Department of Pediatric Hematology-Oncology, IRCCS “Bambino Gesù” Children’s Hospital, Rome, and Department of Pediatrics, University of Pavia, Pavia, Italy; 5 Pediatric Onco-Hematology, Regina Margherita Children’s Hospital, AOU Città della Salute e della Scienza, Turin, Italy; 6 Department of Woman and Child Health, Laboratory of Haematology-Oncology, University of Padova, Padova, Italy; 7 Department of Pediatric Hematology-Oncology, IRCCS “Giannina Gaslini” Children’s Hospital, Genoa, Italy; 8 Pediatric Hematology-Oncology, Ospedale Microcitemico, Cagliari, Italy; 9 Oncoematologia Pediatrica, Fondazione IRCCS Policlinico San Matteo, Pavia, Italy; 10 Department of Cellular Biotechnologies and Hematogy, “Sapienza” University of Rome, Rome, Italy; 11 Pediatric Hematology-Oncology, ‘Spedali Civili’, Brescia, Italy; 12 UOC di Pediatria ad indirizzo Oncoematologico Ospedaliera, Policlinico di Bari, Bari, Italy; 13 Pediatric Hematology-Oncology, IRCCS Meyer Children’s Hospital, Florence, Italy; 14 Pediatric Hematology-Oncology, Azienda Ospedaliera Pugliese Ciaccio, Catanzaro, Italy; 15 Center of Pediatric Hemato-Oncology, Azienda Policlinico - OVE, Catania, Italy; 16 Department of Pediatric Haemato-Oncology, ARNAS Civico e Di Cristina, Palermo, Italy

Osteonecrosis (ON) is a well-known, disabling complication that can occur in pediatric acute lymphoblastic leukemia (ALL), either during treatment or after its discontinuation^1^. Severity of ON may range from asymptomatic to debilitating, causing severe pain, limited motion of joints, and finally joint destruction, thus negatively affecting patients’ quality of life (QoL)^2^. Prevalence and risk factors varied widely in published papers^3^. This wide variability might be related to differences in definition of ON or in detection methods, but also to difference in age of investigated cohorts (e.g. percentage of adolescents) and in type/cumulative doses of steroids employed during treatment^4^.

ON pathogenesis in patients with childhood ALL is not fully elucidated^5^, being presumably multifactorial. Although corticosteroids, both prednisone (PRED) and dexamethasone (DEXA), have been identified as the main cause of ON in children with ALL, causing increased intramedullary pressure and subsequent blood stasis, other drugs, including methotrexate and asparaginase, may contribute to the development of this complication^3,6,7^.

ON incidence correlates with age at diagnosis and female gender, suggesting a contribution of growth and hormonal factors in ON pathogenesis^8^. Hyperlipidemia/hypercholesterolemia, were also observed in patients with ON, but the real meaning of this association requires further investigations^1,9^. Other proposed risk factors include Caucasian race^4^, higher BMI, genetic polymorphisms involving PAI-1 4G/5G, VDR, TYM, CYP3A, ACP1 genes^7^.

In this multicenter study, we assessed incidence of ON, its risk factors and outcome in children with ALL enrolled in two clinical trials conducted in centers affiliated to the Italian Association of Pediatric Hematology and Oncology (AIEOP)^10^.

A cohort of 3691 ALL patients (aged 1–17 years at diagnosis), diagnosed from September 2000 to December 2011 and treated according to either the AIEOP-BFM-ALL-2000 protocol (clinicaltrials.gov/NCT00613457)^10^ or subsequent guidelines (AIEOP-ALL-R2006), was analyzed. Complete information on ON-related symptoms, radiological findings, treatment, and outcome of ON were retrospectively retrieved with ad hoc forms. Infants and Philadelphia positive ALL were excluded from the study because they were enrolled in ad hoc trials. Cases of ON occurring after hematopoietic stem cell transplantation (HSCT) or relapse were not part of this study.

ALL treatment was risk-adapted based on genetic features and cytological/molecular response (see Table 1 for details)^10^.

**Table 1:** Characteristics of the 99 patients developing osteonecrosis (ON) according to age at diagnosis of ALL

Characteristics at ON diagnosis	1–9 years		10–17 years		Total	
	*N*	%	*N*	%	*N*	%
Total no. of episodes	22		77		99	
Phase of onset^a^						
Prephase or 1a	0		3	*3.9*	3	*3.0*
1b	2	*9.1*	3	*3.9*	5	*5.0*
Protocol M	1	*4.5*	2	*2.6*	3	*3.0*
High-risk blocks	0		2	*2.6*	2	*2.0*
Prot II	2	*9.1*	7	*9.1*	9	*9.1*
Prot III	3	*13.6*	10	*13.0*	13	*13.2*
Maintenance	10	*45.5*	38	*49.3*	48	*48.5*
Off therapy	4	18.2	12	15.6	16	16.2
Symptomatic						
Yes	19	*90.5*	60	*82.2*	79	*84.0*
No	2	*9.5*	13	*17.8*	15	*16.0*
NK	1		4		5	
Obesity						
Yes	3	*15.0*	12	*16.7*	15	*16.3*
No	17	*85.0*	60	*83.3*	77	*83.7*
NK	2		5		7	
Thyroid dysfunction						
Yes	0		1	*1.8*	1	*1.4*
No	17	*100.0*	55	*98.2*	72	*98.6*
NK	5		21		26	
Cranial radiotherapy						
Yes	2	*11.1*	11	*16.2*	13	*15.1*
No	16	*88.9*	57	*83.8*	73	*84.9*
NK	4		9		13	
ON extension						
One site	7	*33.3*	23	*30.6*	30	*31.2*
≥2 sites	14	*66.7*	52	*69.4*	66	*68.8*
Interventions for ON						
Arthroplasty						
Yes	0		18	*24.0*	18	*19.4*
No^b^	20	*100.0*	55	*76.0*	75	*80.6*
NK	2		4		6	
Other therapies/interventions^c^						
Yes	9	*42.9*	38	*52.1*	47	*50.0*
No	12	*57.1*	35	*47.9*	47	*50.0*
NK	1		4		5	
Last follow-up						
Asymptomatic	21	*100.0*	67	*90.5*	88	*92.6*
Pain, w/o limitation	0		3	*4.1*	3	*3.2*
Pain, with limitation	0		4	*5.4*	4	*4.2*
NK	1		3		4	

*NK* not known

^a^Treatment was adapted according to the group of risk. In particular, patients with at least one of the following criteria, namely prednisone-poor-response after 7-day PDN pre-phase, no complete remission on day 33, evidence of t(4;11), or minimal residual disease (MRD) value of 5 × 10^−4^ or more on day 78, were allocated to the high-risk (HR) group. In the absence of HR features, patients were allocated to the standard-risk (SR) group if MRD was negative on days 33 and 78. The remaining patients were allocated to the medium-risk (MR) group^10^

^b^Arthroplasty planned in four patients

^c^They included: (i) alternative surgical interventions, such as osteotomy (*n* = 2), tenotomy (*n* = 1), arthroscopy (*n* = 1), core decompression (*n* = 2); (ii) invasive procedures, as hyaluronic acid infiltration (*n* = 1), infiltrations of bone matrix, stem cells and autologous platelet gel (*n* = 3); (iii) noninvasive interventions, as bisphosphonates (*n* = 14), hyperbaric oxygen therapy (*n* = 5), magnetotherapy (*n* = 4), weight-bearing restrictions (*n* = 4), physiokinesitherapy (*n* = 14), or others (*n* = 3) (ultrasound, ionophoresis, and gymnastics); (iv) medical therapy with anti-inflammatory and analgesic drugs (*n* = 6) or Vitamin D (*n* = 2)

ON was suspected in case of joint pain and confirmed by radiological imaging, such as computed tomography (CT) or magnetic resonance imaging (MRI). In some cases, ON was occasionally detected during radiological investigations carried out for other reasons (e.g. trauma). We evaluated site of occurrence and ON extension (unilateral or bilateral), phase of treatment and symptoms at onset, type and cumulative doses of steroids administered, risk factors, such as obesity and thyroid dysfunction, and type of ON-related treatment.

For outcome assessment, clinical symptoms at last follow-up were recorded. Patients were classified as either asymptomatic if pain-free, or symptomatic if they had mild/moderate pain without functional limitations, or severe pain with limitations of daily activities.

Possible correlation between ON occurrence and clinical characteristics/potential risk factors were statistically analyzed. Descriptive statistics and chi-square test for association were used. Cumulative incidence of ON was estimated adjusting for competing risks of failure (resistance, relapse, death, second malignant neoplasm) and compared using the Gray’s test. Patients transplanted in CR1 were censored at HSCT.

Ninety-nine patients (2.7% of the whole cohort) experienced ON, the 5-year cumulative incidence being 2.4% (SE 0.3). At ON diagnosis, 84% of patients were symptomatic. ON affected 47/1631 females (2.9%) and 52/2060 males (2.5%) (*p*-value = 0.5). Median age at diagnosis of ALL in children affected by ON was 13.5 years vs 4.8 years in those not affected (*p*-value < 0.001). The percentage of patients with ON increased progressively with age being 0.6% (12 ON/2180 patients), 1.4% (10 ON/730 patients), 9.0% (52 ON/581 patients), 12.5% (25 ON/200 patients) for the age groups 1–5, 6–9, 10–14, and 15–17 years, respectively (*p*-value < 0.001).

Details of patient- and disease-related characteristics, as well as treatment modalities, are shown in Table 1 for children less than 10 years of age (78.8% of the cohort) or older (10–17 years, 21.2%). Most ON cases (*n* = 77) occurred among the 781 patients aged 10–17 years at ALL diagnosis (Fig. 1a).

**Fig. 1:**
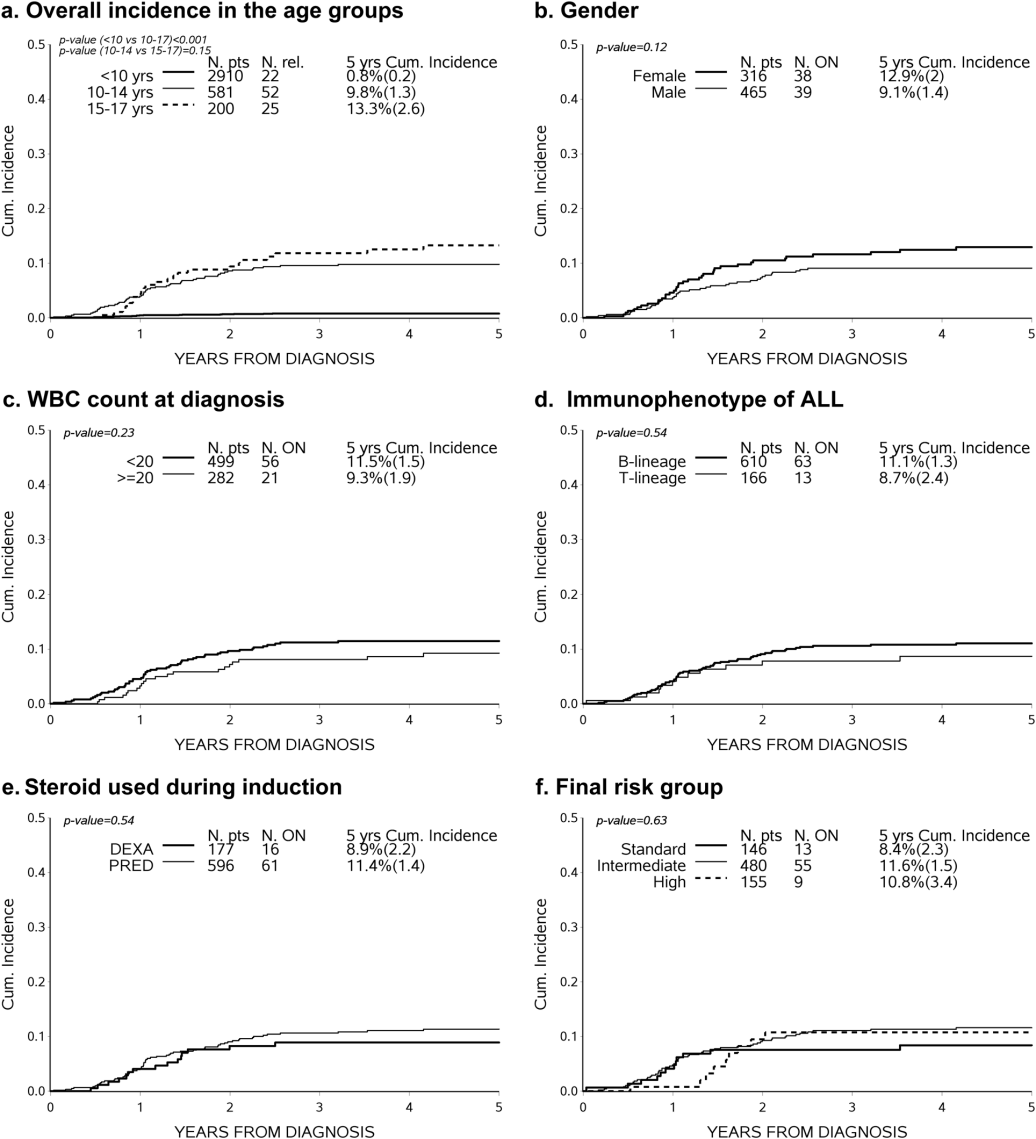
**a** Five-year cumulative incidence of ON according to patient's age at ALL diagnosis. **b**–**f** Five-year cumulative incidence of osteonecrosis in ALL patients aged 10–17 years, according to different characteristics at diagnosis

The 99 ON cases were diagnosed during induction (*n* = 3), consolidation/intensification (*n* = 10), reinduction (*n* = 22) and during maintenance (*n* = 48) or after treatment discontinuation (*n* = 16).

Eighteen ON cases underwent arthroprothesis’ intervention (19.4%), this surgery being planned in 4 additional patients; 47 cases (50%) underwent one or more alternative interventions (see Table 1).

At last follow-up visit, 7.4% of patients were symptomatic, including 4.2% with limitations of daily activities. A single site was involved in one-third of cases; the most frequent sites were hip, knee and ankle. ON (unilateral or bilateral) in these sites was diagnosed in 57%, 57%, and 33% of patients aged 1–9 at ALL diagnosis and in 69%, 49%, and 33% of patients aged 10–17 years at ALL diagnosis, respectively.

Restricting the analysis to patients aged ≥ 10 years, the cumulative incidence of ON was 12.9% in females vs. 9.1% in males (*p*-value = 0.12); no statistical difference was seen when we considered age 10–14 vs. 15–17, WBC count, immunophenotype or risk group stratification. ON incidence was similar also for patients treated with DEXA (8.9%) or PRED (11.4%) during induction and in patients receiving high-risk treatment, despite the higher cumulative dose of steroids administered in this risk group (Fig. 1b–f).

In our large, retrospective cohort study, the ON cumulative incidence was 2.4%. The analysis performed on different age groups highlights that patients 10 years of age or older had a significantly higher ON incidence than those under this age (10.7% vs. 0.8%, respectively, *p* < 0.001), with increasing occurrence in older adolescents (9% in patients aged 10–14 years and 12.5% in patients aged 15–17 years). The peak of incidence in older patients could be related to sexual hormone production during puberty; in our cohort, 62% of ON appeared after puberty.

In literature, the incidence of ON varies widely^2–7,11^, mainly due to the different nature of studies, retrospective (1.8%)^5^ vs. prospective (4.7–7%)^10,12^, or to the diagnostic approach utilized to detect ON. Overall, ON studies confirmed a higher incidence, ranging from 8.9% to 22.7%^5–12^, in patients older than 10 years at diagnosis. A prospective study, which included a routine MRI screening at different time points during treatment and at therapy discontinuation, regardless of symptoms, reported a cumulative incidence of any vs. symptomatic ON of 71.8% vs. 17.6%, respectively; in patients older than 10 years of age at diagnosis, 44.6% developed symptomatic ON compared with 10% in younger patients^7^. Our lower incidence might be explained by the retrospective nature of the study, with a consequent tendency to underreporting.

Although glucocorticoids, especially DEXA, have been considered the main determinants for ON development, the role played by other drugs remains to be fully elucidated^6,12^. In our cohort, ON incidence was not increased in patients given DEXA vs. those receiving PRED during induction phase. By contrast, the Children’s Oncology Group, reported that DEXA during induction has higher risk of inducing ON among high-risk adolescents and young adults (24%) as compared with PRED (16%)^13^. In a subsequent study, the use of alternate-week DEXA during delayed intensification significantly reduced ON incidence (8.7%) as compared to the continuous administration of the drug (17%)^8^.

There is no consensus on the role played by patient gender in ON development. Mattano et al. reported an augmented ON incidence in females (17.4% vs. 11.7% in males; *p* = 0.03);^4^ the same group subsequently confirmed higher ON incidence in girls (17.2% vs. 7.9%) in patients aged 10–21 years^8^. Other studies, including ours, however, did not confirm these findings^5,12^.

In our study population, the peak of incidence was observed during maintenance therapy or after treatment discontinuation (48% and 16%, respectively), this finding being in agreement with other studies reporting a mean time from diagnosis of ALL to that of ON comprised between 1 and 2 years^2,6,11,12^.

There is no consensus on how to manage ON in ALL patients. Different medical therapies have been used, including hyperbaric oxygen, prostaglandins, statins, and bisphosphonates, with variable and inconsistent results^12,13^. Although prophylactic Bisphosphonate seems be an attractive strategy to prevent joint collapse^14^, small lesions could improve spontaneously and the real efficacy of non-surgical intervention is difficult to demonstrate^12^. Arthroplasty should be reserved to severe grade III-IV ON in older patients developing osteoarthritis or deformity of articular surface and subsequently mechanical failure; ideally, the surgical approach should be performed in early adult life, due to durability of current protheses^12^. In our cohort, bisphosphonates were given only to 14 patients; thus, no firm conclusion on efficacy can be drawn. Arthroprothesis was performed in 18 patients with extensive area of involvement (>30%), or lesion sites, such as hip and knee, that could heavily limit daily activities. Since treatment is delayed whenever possible, it is still too early to estimate the final indication to orthopedic surgery.

A recent study, investigating long-term outcome of symptomatic osteonecrosis in ALL children, found that, after a median follow-up time of 4.9 years, symptoms resolved completely in 40% of patients^6^. The natural history of ON in children and the factors influencing long-term outcome are still to be fully elucidated^14^. In addition, no universally adopted scale for outcome assessment, is available, resulting into difficulties comparing studies. In our cohort, at last visit, 92.6% of patients were asymptomatic, 3.2% had persistent pain without functional limitations and 4.2% had pain with limitations of daily activities, confirming possible spontaneous improvement during long-term follow-up^12^.

Although MRI is the gold standard imaging for evaluation of ON^11^, there is no consensus on the use of MRI for screening either in asymptomatic patients or at pain onset. While Nachman^15^ suggested that MRI screening does not provide clinical benefit in asymptomatic cases, Kaste et al. reported that detection of ON lesions in asymptomatic patients afforded opportunities for prompt interventions, to prevent progressive joint damage^11^.

In conclusion, our study confirms that ON in patients treated for ALL is an age-dependent adverse event and that diagnostics and management of ON in these patients continue to present many controversial issues. Future studies on genetic predispositions or risk factors for ON occurrence, prospectively studying long-term consequences on joint mobility, and on QoL, are needed to better identify patients at high risk of this disabling complication and to develop evidence-based guidelines to uniform diagnostic/management approaches.

## References

[1] te Winkel ML, Pieters R, Wind EJ, Bessems JH, van den Heuvel-Eibrink MM (2014). Management and treatment of osteonecrosis in children and adolescent with acute lymphoblastic leukemia. Haematologica.

[2] Girald P (2013). Symptomatic osteonecrosis in childhood leukemia survivors: prevalence, risk factors and impact on quality of life in adulthood. Haematologica.

[3] Kunstreich M, Kummer S, Laws HJ, Borkhardt A, Kunlen M (2016). Osteonecrosis in children with acute lymphoblastic leukemia. Haematologica.

[4] Mattano LA, Sather HN, Trigg ME, Nachman JB (2000). Osteonecrosis as a complication of treating acute lymphoblastic leukemia in children: a report from the Children’s Cancer Group. J. Clin. Oncol..

[5] Burger B (2005). Osteonecrosis: a treatment related toxicity in children with acute lymphoblastic leukemia (ALL)-experiences from trials ALL-BFM 95. Pediatr. Blood. Cancer.

[6] te Winkel ML (2011). Prospective study on incidence, risk factors, and long-term outcome of osteonecrosis in pediatric acute lymphoblastic leukemia. J. Clin. Oncol..

[7] Kawedia JD (2011). Pharmacokinetic, pharmacodynamic and pharmacogenetic determinans of osteonecrosis in children with acute lymphoblastic leukemia. Blood.

[8] Mattano LA (2012). Effect of alternate-week versus continuous Dexamethasone scheduling on the risk of osteonecrosis in acute lymphoblastic leukemia: results from CCG- 1961 randomized cohort trial. Lancet Oncol..

[9] Bhojwani D (2014). Severe hypertriglyceridaemia during therapy for childhood acute lymphoblastic leukaemia. Eur. J. Cancer.

[10] Moriche A (2016). Dexamethasone vs prednisone in induction treatment of pediatric ALL: results of the randomized trial AIEOP-BFM ALL 2000. Blood.

[11] Kaste SC (2015). Utility of early screening magnetic resonance imaging for extensive hip osteonecrosis in pediatric patients treated with glucocorticoids. J. Clin. Oncol..

[12] Padhye B, Dalla-Pozza L, Little D, Munns C (2016). Incidence and outcome of osteonecrosis in children and adolescents after intensive therapy for acute lymphoblastic leukemia (ALL). Cancer Med..

[13] Larsen EC (2016). Dexamethasone and high-dose methotrexate improve outcome for children and young adults with high-risk B-acute lymphoblastic leukemia: a report from Children’s Oncology Group Study AALL0232. J. Clin. Oncol..

[14] Padhye B, Dalla-Pozza L, Little DG, Munns CF (2013). Use of zoledronic acid for treatment of chemotherapy related osteonecrosis in children and adolescents: a retrospective analysis. Pediatr. Blood. Cancer.

[15] Nachman JB (2011). Osteonecrosis in childhood ALL. Blood.

